# The hazards of dealing with response time outliers

**DOI:** 10.3389/fpsyg.2023.1220281

**Published:** 2023-08-24

**Authors:** Ivan I. Vankov

**Affiliations:** Institute of Neurobiology, Bulgarian Academy of Sciences, Sofia, Sofia City, Bulgaria

**Keywords:** outliers, response times, false alarms, researcher degrees of freedom, null hypothesis significance testing

## Abstract

The presence of outliers in response times can affect statistical analyses and lead to incorrect interpretation of the outcome of a study. Therefore, it is a widely accepted practice to try to minimize the effect of outliers by preprocessing the raw data. There exist numerous methods for handling outliers and researchers are free to choose among them. In this article, we use computer simulations to show that serious problems arise from this flexibility. Choosing between alternative ways for handling outliers can result in the inflation of *p*-values and the distortion of confidence intervals and measures of effect size. Using Bayesian parameter estimation and probability distributions with heavier tails eliminates the need to deal with response times outliers, but at the expense of opening another source of flexibility.

## 1. Introduction

Response times (RT) are one of the most widely used dependent measures in cognitive psychology. Analyses of RT are often obscured by the presence of outliers – unusually large or small values which are not produced by the cognitive processes under investigation. Such extreme values can distort the estimated statistics and lead to incorrect statistical inferences. In particular, outliers decrease the power of statistical tests and thus increase the chance of failing to find an existing effect. Therefore, it is a widely accepted practice to preprocess RT data before analyzing them in order to reduce the effect of outliers and to increase the power of the statistical test.

It is important to stress that in this paper we define outliers as values contaminated by adding noise resulting from some random process which is unrelated to the process that we study. Response times can also include genuine (or ‘interesting’, Aguinis et al., 2013; Leys et al., 2019) outliers which could be of theoretical interest. If the presence of such values is predicted, they should by no means be discarded or their effect mitigated.

There is no overall agreement on how to deal with outliers in RT data. Ratcliff (1993) analyzed several popular methods and found that their ability to isolate the influence of outliers depends on a number of factors, such as the exact form of the RT distribution and the prevalence of outliers, and therefore can vary between studies. Ratcliff noted that researchers should decide how they are going to process RTs before conducting the experiment, but it is doubtful that this recommendation is always followed. The abundance of approaches to treating outliers suggests that researchers might be tempted to explore different ways of preprocessing RT data and select to report only the method which leads to statistically significant results supporting their hypotheses. Indeed, a survey among academic psychologists (John et al., 2012) reported that almost half of them admit to have been involved in selective reporting of data such as omitting data points after seeing their impact on the analysis. We will further support the claim that the choice of outlier treatment is often arbitrary and is therefore a potential source for selective reporting by showing that a variety of methods are used in studies investigating the same phenomenon and authored by the same researchers. The goal of this work is to bring attention to the detrimental effects of such a research practice on the correct interpretation of study results. We will show that it considerably increases the rate of false alarms (i.e., the cases in which it is inferred that an effect exists when it is actually absent) and that the problem cannot be avoided by switching to non-frequentist statistical methods, such as Bayesian parameter estimation.

## 2. Evidence for researchers degrees of freedom in choosing how to treat RT outliers

Ulrich and Miller (1994) reviewed the 1992 volume of the “Journal of Experimental Psychology: Human Performance and Perception” and analyzed 35 articles reporting studies of RT. They found that the raw response times were processed without any measures to account for outliers in only about one third of the analyses. In all the other cases the authors used a variety of techniques to reduce the effect of outliers – median aggregation, cutting off data beyond a critical value or a specific number of standard deviations from the mean. Simmons et al. (2011) analyzed about 30 articles in ‘Psychological Science’ and also reported unjustified variability in decisions on how to define and treat outliers.

The availability of various methods for dealing with outliers does not necessarily mean that the choice which one to apply in a particular study is arbitrary and a “potential fodder for self-serving justifications” (Simmons et al., 2011). It is possible that certain methods are preferred for particular study designs or cognitive processes, either because they are known to be effective in these situations or because of an established tradition of unknown origin. In either case, it would be unfair to presume that authors misuse the availability of alternative ways to process their data. To rule out such a possibility, we decided to investigate the choice of methods for treating outliers in studies which all investigate the same phenomenon - the Stroop effect (Stroop, 1935).

We searched the PsycNet database for articles published in “Journal of Experimental Psychology: Human Perception and Performance” and having the keyword ‘Stroop’ in their description, while limiting the scope of the search to publications dated between the year of 2000 and 2020. Thirty five papers were found^1^, only one of them not reporting response times data (it was a correction). By reviewing the methods section, we identified twenty five different methods for dealing with outliers (Appendix A), Only four papers did not report any treatment of response time outliers. The majority of studies trimmed response times above or below specific cut-off values or a certain number of standard deviations. The upper cut-off value ranged across studies between 1,000 ms and 4,000 ms (m = 2,109 ms) and the percentage of removed data points (when reported) varied between 0.06 and 6.60% (m = 2.24%). There was just one article applying more than one method, but we identified seven cases in which the same first (2 cases) or last (5 cases) author was involved in papers using different methods to treat outliers in a Stroop task. On the contrary, there were just two cases in which papers having the same first or last author stuck to the same method. In order to further explore the extent to which authors are willing to explore different methods, we checked whether the first authors of the papers in our sample have authored publications about the Stroop effect in other journals in the PsycNet database. We found such publications for seven authors and only three of them showed consistency in treating outliers across studies.

Overall, our analysis revealed that there is considerable variability in how researchers choose to handle responsive times outliers even in studies sharing similar designs and research questions. In none of the papers reviewed the choice of method was empirically or theoretically justified. This seems to bother neither authors, nor reviewers or editors, given that we limited our review to articles published in a single journal and within a relatively short time frame. However, it is important to stress that the variability of methods does not automatically entail that some researchers are engaging in questionable practices as it could be attributed to other factors, such as evolving laboratory practices.

## 3. Simulations

Below we present a series of simulations showing how the freedom to explore different methods for dealing with outliers and to select one based on the results can affect the interpretation of an experimental outcome. The first simulation is a replication of Ratcliff (1993) work which outlines the importance of taking measures to treat outliers in order to recover the statistical power of the study. Simulation 2 demonstrates how the analysis of the mean difference between two samples of response times can be compromised if researchers explore several methods to handle outliers and select one which leads to a statistically significant difference between conditions. In Simulation 3 we show that descriptive statistics (effect sizes and confidence intervals) can also be distorted by this practice. The last simulation reveals that a more advanced statistical method - Bayesian parameter estimation - can eliminate the need to deal with outliers, but at the expense of opening other sources of flexibility for alternative interpretation of the data.

### 3.1. Implementation

The setup of the simulations closely follows Ratcliff (1993). Simulated response times are sampled from a convolution of a normal and an exponential distribution (also known as Ex-Gaussian distribution), which is particularly suited for modeling response times distributions (De Boeck and Jeon, 2019; Tejo et al., 2019). However all of the results can be replicated by sampling response times from a normal distribution.

The simulated RT experiments had two experimental conditions, 10 observations per condition and 30 subjects. Response times were generated by the following formula:


RT∝N(μ,σ)+exp(λ)+B(p)·U(a,b)

**Figure 1:**
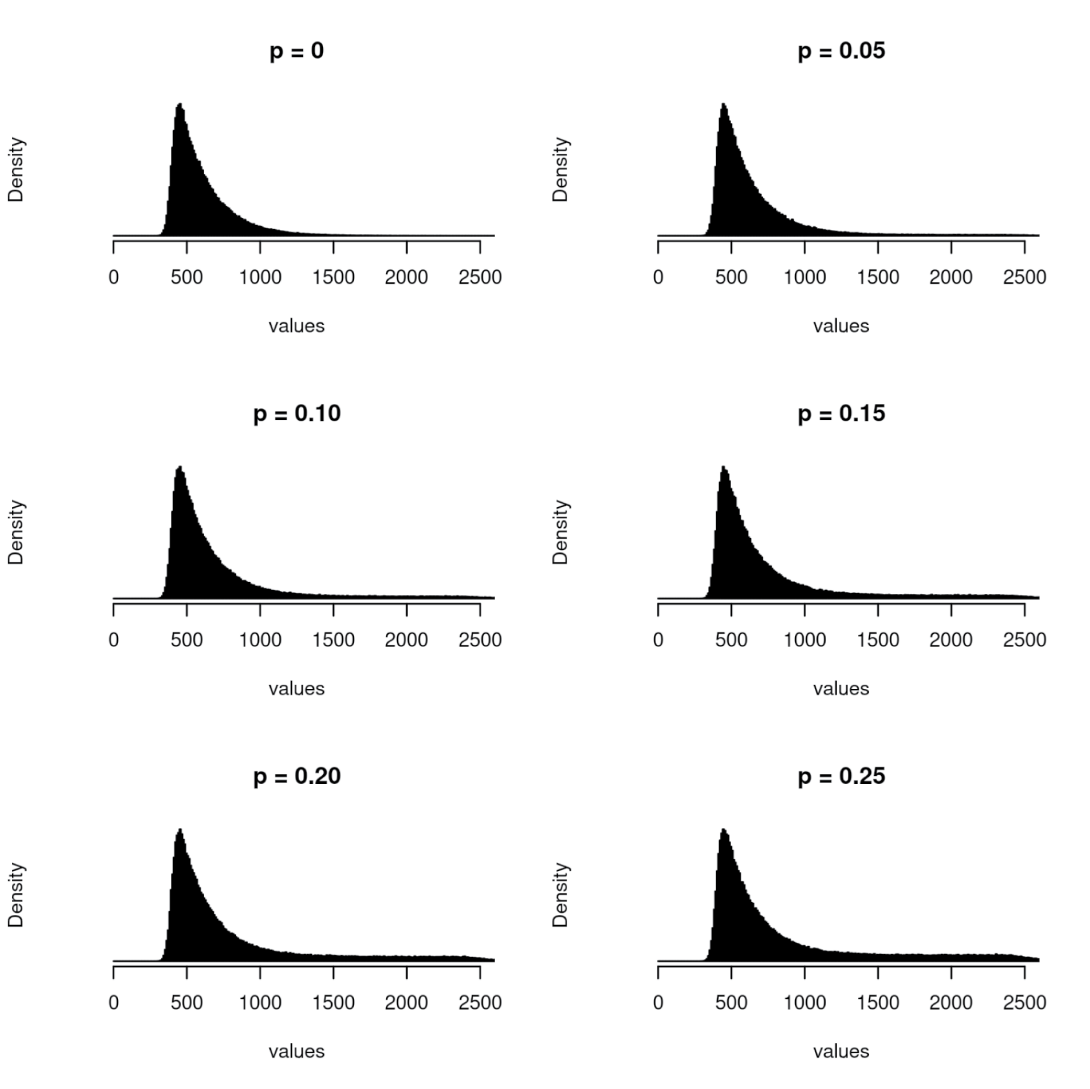
Empirical distribution of simulated data as a function of the proportion of outliers (*p*) - data contaminated with random noise. Note the right skew which is typical of the distribution of response times.

Where *N*, *Exp, B and U* are, respectively, a normal, an exponential, a Bernoulli and an uniform random variable. The parameters of Ex-Gaussian components were the same as in Ratcliff (1993) and were kept constant across simulations: μ = 400, 𝜎 = 40, λ = 200. An effect size was simulated by adding a constant to the mean of normal distribution in one of the experimental conditions. The presence of outliers was also modeled following Ratcliff (1993) by adding noise to some of the response times. The noise was sampled from a uniform random distribution ranging from 0 to 2000 (a = 0, b = 2000). The proportion of response times, to which noise was added, was controlled by *B* (*p*), which took a value 1 with probability *p* and 0 with probability (1 – *p*). Figure 1 shows the distribution of the simulated data as a function of *p*. The particular values chosen for the parameters of the simulations are not representative of any empirical phenomenon but result in simulated data which is typical for research involving response times. We did not explore other parameter setups and therefore the results obtained cannot be generalized to datasets with a qualitatively different distribution of response of times without running further simulations. Morís Fernández and Vadillo (2020) conducted a similar analysis by simulating several different distributions of response times and found a similar pattern of results as us, suggesting the choice of distribution parameters is not critical.

All of the simulations were based on the same study design – a within-subject experiment with two experimental conditions. For each simulation run we generated 600 random data points (30 subject × 2 conditions × 10 data points per condition). The results of the simulated experiment were analyzed by aggregating (i.e., averaging unless the method of aggregation by median was used to treat outliers) the data by subject and performing a pair-wise Student’s *t*-test. This procedure was repeated 10,000 times for Simulation 1–3 and 1,000 times for Simulation 4 and then averaged before presentation. An additional simulation in Appendix B shows the choice of sample size (i.e., number of simulated subjects) and the number of observations per subject per condition was not critical for the results obtained in Simulation 2 - the same pattern of results was obtained when the number of data points per condition varied between 10, 30, 50 and 89, and the number of simulated subjects varied between 10, 20, 30 and 40.

The simulations were programmed in Python and R and all the code is available to download at https://osf.io/xn4cz.

### 3.2. Outlier treatment methods

It is possible to group the existing methods for dealing with outliers into three categories. The first approach is to run the analysis on the medians of the collected RTs, rather than the means. The rationale for using this method is that the median is a measure of central tendency which is less sensitive to the presence of extreme values. Second, it is common practice to transform the raw RT data in order to reduce the effect of extreme values. The most widely used transformations are the logarithmic (f(x) = log(x)) and the inverse transform (f(x) = x^−1^) functions. The advantage of this method is that no data is lost and the resulting distribution is closer to the normal one. The third and most widely used approach is to truncate values which are below or beyond a given threshold. There are numerous ways to define the threshold, either by choosing an arbitrary cut-off value or by setting it at a fixed number of standard deviations from the mean. There are also variations of the definition of the mean – it could be the grand mean (i.e., the mean of all the values in the sample), the mean of an experimental condition, the subject mean, or the subject mean per condition. Another source of variation concerns the treatment of removed data points –they can either be left out, leading to loss of data, interpolated or replaced with the most extreme values allowed.

Ratcliff (1993) showed that none of the above-mentioned procedures has a clear advantage and therefore it is up to the researcher to decide how to treat outliers in a particular study. Twenty particular methods have been selected for the subsequent simulations (Table 1). Their choice was justified by our survey of methods used to treat outliers in papers investigating the Stroop effect, which showed that the majority of authors chose to trim values beyond a certain threshold. The particular cut-off values in Table 1 were motivated by the observation that researchers prefer to use round numbers for that purpose. We also included two of the methods recommended by Ratcliff which preserved the number of observations - the logarithmic and the inverse transformation. It should be noted that there exist many more legitimate ways to treat response times outliers. The list in Table 1 is by no way comprehensive but even this limited set of methods is enough to demonstrate the hazards of being able to choose among alternative methods to process response times.

**Table 1:** Methods for dealing with outliers.

Method	Description	Type
1	Leave data as it is	Ignore
2	Run analyses on median subject RTs	Median
3	Transform raw data by using the logarithmic function	Transform
4	Inverse transform raw data	Transform
5–7	Exclude values which are more than 2, 2.5 or 3 standard deviations below or above the experiment mean response time.	Truncate
8–11	Exclude values which are more than 1.5, 2, 2.5 or 3 standard deviations below or above participants’ mean response times.	Truncate
12–14	Exclude values which are more than 2, 2.5 or 3 standard deviations below or above the mean response time per condition.	Truncate
15–20	Exclude values lower than 100 and larger than a fixed cut-off value (800, 1,000, 1,200, 1,500, 1750 or 2000)	Truncate

### 3.3. Simulation 1: the effect of outliers on statistical power

Before exploring the consequences of choosing between alternative methods for processing response times, it is important to demonstrate why one would like to do this at all. The framework of null hypothesis significance (NHST) testing defines two types of errors that can be committed when running a test of statistical significance. A Type I error, also known as a false positive or a false alarm, is committed when one rejects a null hypothesis which is actually true. For example, finding a statistically significant difference between the means of two samples of data which have been drawn from the same distribution would be a Type I error. On the contrary, a Type II error (failing to reject a null hypothesis when it is false or a false negative) would be committed if we fail to find a statistically significant difference between the means of samples which come from distributions with different means. The statistical power of a test is defined as the probability of not committing a Type II error, i.e., how likely it is to detect a real effect.

**Figure 2:**
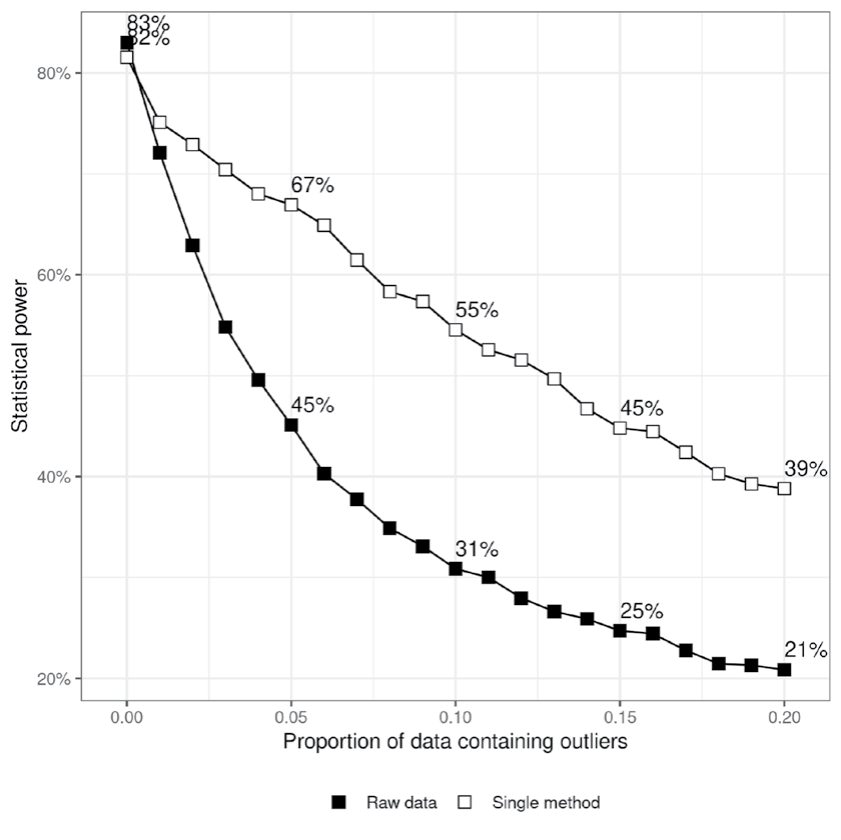
Statistical power as a function of the proportion of data containing outliers. The upper series shows power when a single, randomly chosen, method for dealing with outliers is applied to the data in each simulation run and the results are averaged. The difference between the two lines indicates the benefit of treating outliers. The criterion for statistical significance is *p* < 0.05.

Figure 2 shows the relation between statistical power and the quantity of outliers present in the sample. In order to generate the data presented in Figure 2, a real difference between the two experimental conditions is modeled by adding a constant (𝜇_diff_) to the mean of the normal component of the RT probability density function. Thus, the RTs in the two conditions are drawn from two different distributions:


$$RT_{1}\propto N\left(400,40\right)+\exp\left(200\right)+B\left(p\right)\cdot U\left(0,2000\right)$$

$$RT_{2}\propto N\left(400+\mu_{diff},40\right)+\exp\left(200\right)+B\left(p\right)\cdot U\left(0,2000\right)$$

The amount of outliers is modeled by *p* - the proportion of RTs to which random noise is added.

Statistical power was calculated by setting 𝜇_diff_ = 50 and running the simulated study multiple times and counting the proportion of cases in which a statistically significant difference was found at the conventional level of statistical significance *α* = 0.05.

The results show that statistical power critically depends on the number of outliers. The test rejects the null hypothesis more than 80% of the time when there were no outliers and its performance drops to 40% when 20% of the response times are potential outliers. This is one of the reasons which motivates researchers to come up with procedures for dealing with outliers in order to restore the power of their experiments. Figure 2 also shows that randomly choosing one of the methods for treating outliers listed in Table 1 may significantly decrease the probability of committing a Type II statistical error. It is not surprising that, knowing the effect of outliers on statistical power, researchers are tempted to try different ways of processing their data in order to minimize the chance of failing to find a real effect.

**Figure 3:**
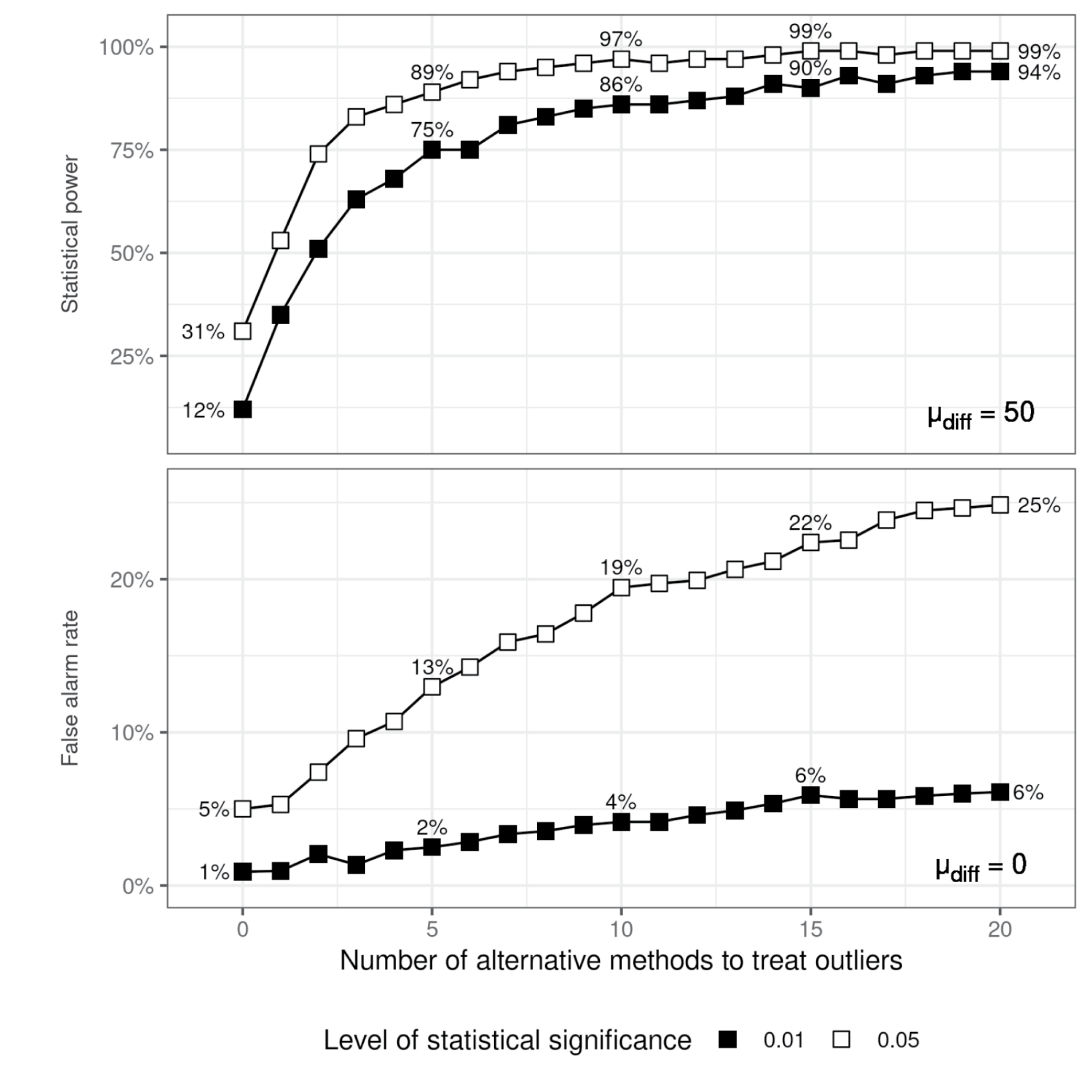
Statistical power (top) and false alarm rates (bottom) as a function of the number of alternative methods for dealing with outliers that have been tried. When estimating statistical power, an effect size was simulated by setting 𝜇_diff_ to 50. The proportion of data containing outliers was fixed to 0.1.

The top panel of Figure 3 demonstrates that the power of an experiment with 10% potential outliers can be fully restored if one explores alternative ways to pre-process the data. To simulate such a procedure, a random subset of the methods from Table 1 was generated and it was checked whether a significant difference was found after applying any of the methods from the subset. The figure shows that choosing between only four different methods for dealing with outliers may increase power from 31 to 86%. The problem is that in doing so we can also increase the chance of ‘revealing’ an effect which is actually not present.

### 3.4. Simulation 2: the effect of outliers on false positives

Ratcliff (1993) explored in detail how various methods for dealing with outliers work to improve statistical power. He noted that some methods were more effective than others in certain situations, but that none of the methods ever affected false alarm rates. However, what happens if we explore several methods and choose the one which leads to a desirable outcome of our particular study? The bottom panel in Figure 3 shows the probability of committing a type I statistical error as a function of the number of methods tried. The figure was generated by assuming that the null hypothesis is true (𝜇_diff_ = 0) and the response times in the two experimental conditions are drawn from the same distribution:


RT∝N(400,40)+exp(200)+B(0.1)·U(0,2000)

The probability of committing a type I error is calculated by repeating the simulated experiment multiple times and counting whether a significant difference was found after applying any of the methods.

Figure 3 demonstrates the hazardous effects of trying to obtain statistical significance by exploring multiple ways to handle RT outliers. Just choosing between three methods doubles the false alarm rate and considering six such methods triples it. Adopting a stricter significance criterion does not help avoiding this problem – it is still possible to get to the desired level of statistical significance. More generally, lowering the level of statistical significance is not a solution to the problem of reliability of results in psychological studies (Trafimow et al., 2018).

It is sometimes argued that the publishing of false findings can be prevented by using large samples. For example, Simmons et al. (2011) suggested that researchers should collect at least 20 observations per cell in order to make sure that the obtained statistically significant differences are not due to statistical and data processing tricks. While this is no doubt a wise recommendation and it certainly always makes sense to collect as much data as possible, it is not possible to address the problem of dealing with outliers in this way. In order to address this issue, we re-ran Simulation 2 by systematically varying the number of simulated subjects (10, 30, 50, 80) and observations per cell (10, 20, 30, 40). The results (Figures A6, A7 in the Appendix B) reveal the same pattern of results as in the bottom panel of Figure 3, indicating that collecting more data cannot prevent generating false positives by selecting among multiple ways to handle RT outliers.

### 3.5. Simulation 3: confidence intervals and effect sizes

Many authors have argued that *p*-values are a poor way to describe the outcome of an experiment and they should be replaced or complimented by reporting confidence intervals and measures of effect size (Cohen, 1994; Hunter, 1997; Fritz et al., 2012; Trafimow et al., 2018). A confidence interval (CI) is a range of values specific to a study, which will contain the true value of a population parameter in a certain proportion (usually 95%) of the times the study is repeated. For example, if we are interested in the difference between the means of two conditions and repeat the study multiple times, in 95% of the replications the corresponding 95% CI will contain the true difference between the conditions. Confidence intervals are computed using the same assumptions and logic as *p*-values and they can be used to make the same statistical inferences. For example, if the 95% CI of the difference between the means of two experimental conditions does not contain 0, then it means that if we reject the null hypothesis, we will be wrong in less than 5% of the cases (i.e., *p* < 0.05). Therefore, the effect of increasing the researchers’ degrees of freedom on producing statistically significant but false results using CIs will be the same as when using *p*-values.

The main advantage of confidence intervals is that, unlike *p*-values, they provide information about the magnitude of the effect and its direction and draw the readers’ attention to the inherent uncertainty of the sample estimate. A large confidence interval indicates low precision of the study and questions its conclusiveness even if a statistically significant result was found. Therefore it is important to know that the width of confidence intervals can be subjected to the same kind of manipulations as *p*-values.

**Figure 4:**
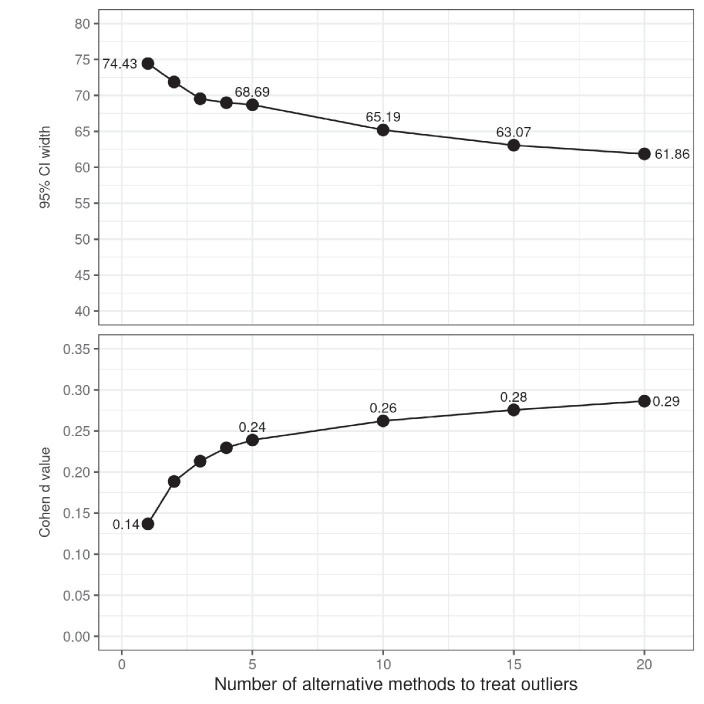
The effect of having flexibility in choosing how to treat outliers on confidence interval width (top) and effect size (bottom). The top panel shows the minimal width of the 95% of the confidence as a function of the number of alternative methods to treat outliers and indicates the extent to which it is possible to exploit researchers’ degrees of freedom to present results as more conclusive than they really are. The confidence interval was calculated only when the difference between conditions was statistically significant at the 0.05 level. The simulation exploring the effect of the number of methods to treat outliers on confidence intervals and effect size were run independently of each other.

Simulation 3 aims to illustrate the extent to which one can selectively minimize the width of a confidence interval by choosing between alternative ways to handle RT outliers. The settings of the simulation are the same as in Simulation 2, but this time the criterion for choosing a particular method is that it not only leads to statistical significance, but also minimizes the width of the 95% CI of the means difference. The outcome of the simulation is presented in Figure 4. The results imply that it is indeed possible to tweak the range of the confidence interval and thus to present the results of the study as more conclusive than they really are.

One of the disadvantages of using *p*-values to describe the outcome of an experiment is that it does not quantify the magnitude of the observed effects. Given that in practice a point null hypothesis (e.g., one which states that the difference of means is exactly 0) can never be true, one may argue that a failure to find a significant result is only an indication of using a small sample (Cohen, 1994). More importantly, it is often the case that researchers are interested not only in whether there is a reliable difference between experimental conditions, but also in how large the effect is. Therefore, most psychological journals nowadays urge authors to include measures of effect size in their statistical analysis. An additional advantage of reporting effect sizes is that they facilitate subsequent meta-analyses and power estimations (Fritz et al., 2012).

The most popular measure of effect size is Cohen (1992), which is used to quantify the difference between two means in terms of number of standard deviations. Figure 4 displays the results of a simulation which aimed to check to what extent it is possible to maximize the absolute value of the Cohen’s *d* by choosing among alternative methods for processing response times data sampled from the same random distribution. The results indicate that it is possible to squeeze an effect size of up to one third of a standard deviation from random data. Given that there are no firm standards about the levels of effect size which are acceptable (Cortina and Landis, 2011; Fiedler, 2011) and that RT experiments often involve subtle manipulations of the independent variable, this finding suggests that reporting effect sizes is not a remedy for the problem at hand.

### 3.6. Simulation 4: modeling outliers with Bayesian parameter estimation

We have shown so far that there are serious problems with considering several methods for dealing with RT outliers and choosing the one to use based on the results, a practice which results in distorted statistical analyses and increased rate of publishing false positive findings. Moreover, there are arguments against the very idea of pre-possessing response times prior to analyzing them (Miller, 1988; Ulrich and Miller, 1994). It is tempting to conclude that the proper way to deal with outliers is not to deal with them at all. However, the results of Simulation 1 demonstrate that working with raw RT data containing outliers can drastically reduce the power of our studies, which is a serious problem by itself. Why do outliers affect statistical power? If an experiment is properly designed and conducted, then random RT outliers will be equally distributed across conditions and will not affect the differences between means. However, outliers increase the pooled standard deviation and thus lead to decreasing the estimates of test statistics such as *t* and *F* values (Rousselet and Wilcox, 2020).

Kruschke (2013) proposed that the frequentist Student *t*-test should be replaced by Bayesian parameter estimation as a tool for comparing metric data coming from two groups. In this statistical framework, researchers assume that their data is being sampled from a set of interrelated random distributions with unknown parameters and the goal is to find the most credible values of these parameters given the data at hand. For example, in order to compare two samples of collected data, one may assume in both cases the data comes from a normal distribution, N_A_(μ_A_, σ^2^) and N_B_(μ_B_, σ^2^), having the same variance. The only difference between conditions, if any, is in the difference between the means of the two distributions (δ = μ_A_ − μ_B_). Using the Bayes theorem and sampling algorithms, such as Markov chain Monte-Carlo, it is possible to estimate the distribution of the most credible values of δ and decide whether and to what extent the evidence supports a hypothesis claiming that there exists a genuine difference between the experimental conditions.

One of the merits of this approach is that it lets researchers model their data more closely by choosing suitable probability density functions. In particular, Kruschke (2013) showed how one can accommodate outliers by describing the data with a *t*-distribution which can have taller tails than the normal distribution. Krushcke however did not show how much the statistical power of the test benefits from using a better model of the data. We addressed this point by conducting a series of simulations of Bayesian parameter estimation with varying model assumptions. We used the code provided by Kruschke (2013), but made several changes in order to facilitate the simulations and to enable a fair comparison with the performance of a *t*-test. The main difference from Kruschke’s model is the assumption that the two groups have the same standard deviation, which decreases the number of parameters by one and speeds up simulations. The priors of the group means were also changed to make them favor the alternative hypothesis to a lesser extent and make the test comparable to a frequentist Student *t*-test. Importantly, these changes do not change the idea of the test originally proposed by Kruschke (2013), nor do they undermine its efficiency or reliability in any way.

Four different probability density functions were used to model the distribution of response times – *t*, *gamma*, *log-normal* and *normal*. Simulated data were generated in the same way as in Simulation 1, but we also varied the number of observations per cell (*n*_obs_) and the mean difference between conditions (𝜇_diff_) in order to make sure that the pattern of results is not specific to a particular number of observations per condition. The proportion of data containing outliers varied between 0 and 0.2. The simulation was repeated 1,000 times and a Bayesian parameter estimation analysis was performed under each of the four distributional assumptions. Statistical power was estimated by calculating the proportion of cases in which the 95% highest density interval (HDI, the narrowest interval containing 95% of the data) of the posterior probability of the difference of means excluded zero.

**Figure 5:**
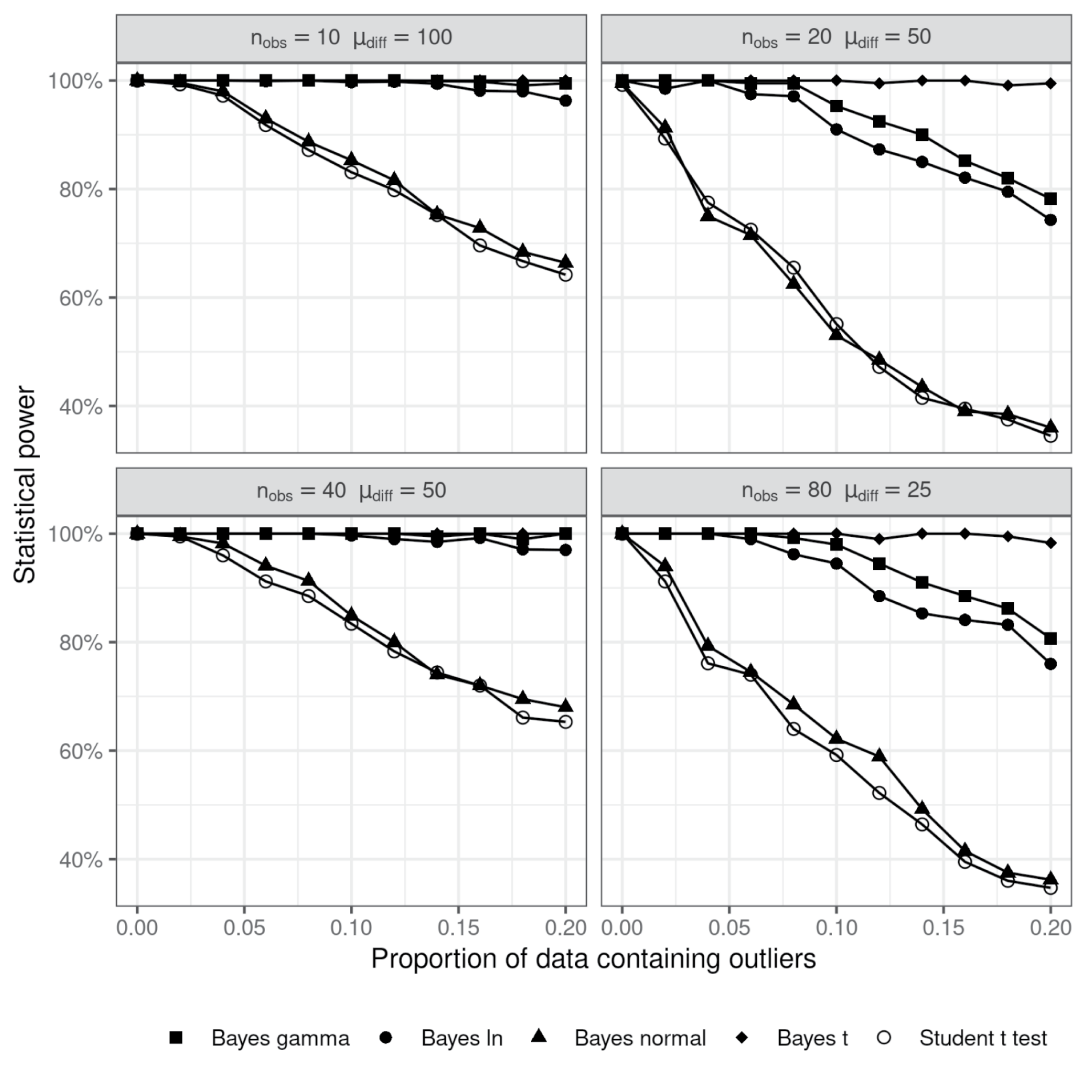
Statistical power of Bayesian parameter estimation as a function of the proportion of outliers and the number of observations per cell. The level of significance for the *t*-test was 0.05.

The results of Simulation 5 are displayed in Figure 5. The first important observation to make is that almost no difference was found between the statistical power of a Student’s *t*-test and Bayesian parameter estimation which assumes RTs are normally distributed. However, the performance of the other versions of Bayesian parameter estimation shows clearly that the negative effect of outliers on statistical power can be overrun by modeling RTs with a distribution function with heavier tails. It is striking that, even with 20% of the data contaminated by noise, all the three distributions which allowed for more extreme values (*t*, *log-normal* and *gamma*) retained near perfect statistical power.

The simulations revealed that it is possible to overcome the problem of analyzing RT data without having to choose between alternative methods for dealing with outliers. Researchers should not try to remove outliers but accommodate them in their statistical models and work with raw data. Although it is possible to do this within the paradigm of classical Fisherian statistics (e.g., Heathcote et al., 1991; Marmolejo-Ramos et al., 2015; Stasinopoulos et al., 2018; Marmolejo-Ramos et al., 2023) as well as with other Bayesian approaches (e.g., Umlauf et al., 2018), the Bayesian parameter estimation approach provides a particularly elegant and straightforward solution while having many other advantages at the same time (Kruschke, 2010).

Could Bayesian statistics also provide a clue to the more general problem of researcher’s degrees of freedom? Simmons et al. (2011) argued that replacing frequentist statistical methods with Bayesian ones can make things even worse as the latter provide more flexibility which authors could exploit to push their analyses in the desired direction. Indeed, our simulations show that there are at least three efficient ways of describing RT data containing outliers, which are equally justifiable.

An additional simulation was conducted to check whether the flexibility in choosing how to model RTs can affect the rate of producing false alarms. To this end, we measured the performance of Bayesian parameter estimation but this time the data in both groups was sampled from the same distribution, as in Simulation 2. For each simulated experiment, it was checked whether any of the alternative Bayesian models produces a 95% HDI of the posterior distribution of the group mean difference which excludes 0. The results are presented in Table 2. The false alarm rates of individual models were similar or below the conventional frequentist level of statistical significance, which means that their superior statistical power and greater ability to handle outliers in comparison to the *t*-test was not at the expense of favoring the null hypothesis. In particular, the gamma distribution seems to be particularly suitable for handling outliers, as it achieves high statistical power while keeping the false alarm rate very low. However, the results also show that having the option to choose between several models of the data can significantly increase the chance of producing a false alarm. In other words, eliminating the flexibility arising from exploring different methods of removing outliers by including them in the statistical model opens another source of flexibility - how exactly to model them. In fact, the flexibility provided by Bayesian statistics can be far greater if we consider the innumerable possible ways of setting up the priors, as well the alternative ways of interpreting the posterior probabilities. The ability to control the prior probabilities of the hypothesis being tested is one of the major virtues of Bayesian statistics, but unfortunately it does not always become immediately clear how much a certain prior favors a hypothesis. Taken together, the results of the simulations described above suggest that merely switching from frequentist to Bayesian statistical methods will not solve the problem of misusing researchers’ degrees of freedom.

**Table 2:** False alarm rates of four versions of Bayesian parameter estimation.

Model	False alarm rate
Bayes normal	5.45%
Bayes ln	5.85%
Bayes gamma	0.25%
Bayes *t*	6.45%
Cumulative	12.12%

The cumulative false alarm rate is calculated by checking whether any of the methods can reject the null hypothesis. The proportion of data containing outliers is fixed to 0.1.

## 4. Discussion

We showed in a series of computational studies that there exist serious hazards related to the analysis of response times containing outliers. On the one hand, the presence of outliers affects statistical power. The problem of low statistical power is not simply that it increases the chance of failing to find a real effect. The findings of underpowered studies are harder to replicate which leads to confusion in the scientific community as it is not possible to determine whether a replication has failed because the original study had produced a false alarm or because its power had been low (Vankov et al., 2014; Zwaan et al., 2017). On the other hand, there are plenty of widely used methods for pre-processing response times which minimize the effect of outliers. There are no rules prescribing which method to use in a particular situation and it is left to the author’s discretion to decide. In effect, researchers are free to explore a number of alternative ways of handling outliers and only report the one which leads to analyses supporting the desired experimental outcome. Our simulations show that the hazards of this practice can be as serious as increasing the expected false alarm rate more than four times. Reporting confidence and effect sizes can alleviate the problem to some extent, as long as they provide additional information about the uncertainty and magnitude of the effects observed, but cannot solve it entirely as these additional measures can also be affected by the flexibility in processing RT data.

The current study considered only a limited set of methods for dealing with outliers, which have been selected based on Ratcliff (1993) and their prevalence in the analyzed sample of papers investigated the Stroop effect. There exist many other, more robust, methods for handling outliers (e.g., Yang et al., 2019). While we do recommend readers to make use of the advances in state-of-the-art research on outlier detection, we would like to stress that the problem discussed in this paper is not specific to any particular method or set of methods. In fact, the more alternative ways to process their data researchers have in their arsenal, the easier it would be for them to bias their analysis in a desired direction.

The Bayesian parameter estimation method proposed by Kruschke (2013) offers a solution to the problem of analyzing response times by letting researchers accommodate outliers in their statistical models. Simulation 4 showed that the negative effect of outliers on statistical power can be avoided if we use a model which fits better the distribution of response times. Unfortunately, the versatility of this approach, which underlies its success in resolving the issue with outliers, has its down side as well - it is possible to try various ways of modeling the data and only report the one which leads to a desired outcome of a study. Nevertheless, the Bayesian parameter estimation way of dealing with outliers has one major advantage compared to the other methods considered in this paper – it forces authors to explicitly state their model assumptions and makes the researchers’ degrees of freedom transparent to the reader. Moreover, unlike frequentist analyses based on *p*-values, the Bayesian approach to statistics does not necessarily result in making a dichotomous decision about the relationship between data and the theory being tested. It is possible that researchers will be less willing to exploit researcher’s degrees of freedom if they are not pressed to come up with an unequivocal verdict about the outcome of their study. We therefore strongly recommend the use of parameter estimation for analyzing response times but urge researchers to bear in mind that it is also susceptible to exploitation of researchers’ degrees of freedom.

The problem of selective reporting and choosing among alternative ways of processing data is already a widely recognized problem in psychology (Simmons et al., 2011; Wicherts et al., 2016). Morís Fernández and Vadillo (2020) identified the treatment of response time outliers as a particular source of researchers’ degrees of freedom which increase false alarm rates by up to 17%. Our results render further support for this claim and elaborate its implications beyond the paradigm of null hypothesis significance testing. First, we provide indirect empirical evidence that such a problem exists by demonstrating the wide repertoire of methods for dealing with outliers which are used to analyze the same phenomenon. Second, we draw attention to the fact that exploiting the researchers’ degrees of freedom in outlier treatments can not only inflate p-values, but can also produce narrower confidence intervals and larger effect sizes when no effect exists. Finally, we show how the problem with response time outliers can be addressed by using Bayesian parameter estimation which eliminates the need to remove or transform any data but opens another source of flexibility which can potentially undermine the credibility of published research.

In our view, the best way to counteract the publishing of false positive findings is by stimulating authors to include as many details about their studies as possible and by fostering critical attitude in reviewers and readers. It is important to understand that the analyses of empirical data are always affected to some extent by the researcher’s beliefs and expectations and care must be taken to make these biases transparent and to reveal their impact on the conclusions drawn (Bishop, 2020). Pre-registering methods and statistical analysis and multiverse analysis might help to prevent unintentional abuse of researcher’s degrees of freedom (Steegen et al., 2016; Nosek et al., 2018; Leys et al., 2019). Last but not least, the distortion of statistical analyses should be addressed by increasing the overall statistical competence of researchers and making them aware of the pitfalls of specific research practices.

## Data availability statement

The datasets presented in this study can be found in online repositories. The names of the repository/repositories and accession number(s) can be found in the article/Supplementary material.

## Author contributions

The author confirms being the sole contributor of this work and has approved it for publication.

## Conflict of interest

The author declares that the research was conducted in the absence of any commercial or financial relationships that could be construed as a potential conflict of interest.

## Publisher’s note

All claims expressed in this article are solely those of the authors and do not necessarily represent those of their affiliated organizations, or those of the publisher, the editors and the reviewers. Any product that may be evaluated in this article, or claim that may be made by its manufacturer, is not guaranteed or endorsed by the publisher.

## Footnotes

^1^All the data related to the literature review is available at https://osf.io/umtgp/.
