# Supplementary material for: The hazards of dealing with response time outliers
Source: Front Psychol. 2023 Aug 24;14:1220281. doi: 10.3389/fpsyg.2023.1220281 (PMC10484222; doi:10.3389/fpsyg.2023.1220281)
Supplement: Supplementary file 1 [file Data_Sheet_1.pdf]

Appendix A

Table 3. Methods for dealing with outliers found in our review of Stroop studies in JEP: HPP published during 2000-2020. The last column indicates the number of articles using the given method.

id	Description	Articles
1	Not mentioned or using raw data	4
2	Recursive data-trimming procedure, Van Selst & Jolicoeur,1994	2
3	Remover RTs shorter than 200 ms and use median RT	1
4	Remove RTs for those trials with latencies straying more than 3 standard deviations from the individual mean, computed for each block and participant	1
5	Remove RTS slower or faster than 2.5 standard deviations from the grand mean of each color	1
6	Remove RTs faster than 100 ms or slower than 1,200 ms	1
7	Remove RTs greater than 3 standard deviations from each individual's mean RT for a cell	1
8	Remove RTs below 200ms or 3 standard deviations below the condition mean or above 2,000 ms or 3 SDs above the condition mean	1
9	Remove RTS shorter than 200 ms or longer than 1500 ms	1
10	Tukey's criterion (i.e., values below the first quartile minus 1.5 times the interquartile range or above the third quartile plus 1.5 times the interquartile range)	1
11	Remove RTs larger than 2,000 ms or smaller than 200 ms	1
12	Remove RTs greater than 2 SDs from the cell mean	1
13	Use median	2
14	Remove RTs shorter than 100 ms or longer than 2,000 ms	1
15	Remove RTs that were 3 standard deviations either above or below the mean	3
16	Remove RTs less than 200 ms or greater than 3,000 ms	3
17	Removed RTs exceeding 2 standard deviations on either side of the mean	1
18	Removed RTs slower than 250ms and faster than 2,500 ms	1
19	Outliers were controlled with a procedure reported in Schmiedek, Oberauer, Wilhelm, Su, and Wittmann (2007). Participant's condition means were calculated, and trials with response times below 200 ms and above 4 standard deviations of the mean were excluded. This procedure was repeated until no further outliers were detected	1
20	Remove RTs less than 200 ms and greater than 1200 ms	1
21	Remove RTs less than 200 ms and greater than 1800 ms	1
22	Remove outliers which stand beyond the absolute deviation around median (Leys, Ley, Klein, Bernard, & Licata, 2013)	1

23	Remove RTs slower than 4000ms	1
24	Remove RTs less than 300 ms and greater than 3000 ms	1
25	Remove RTs less than 200 ms and greater than 1000 ms	1

Appendix B

Simulation B1: The effect of sample size and the number of observations per cell

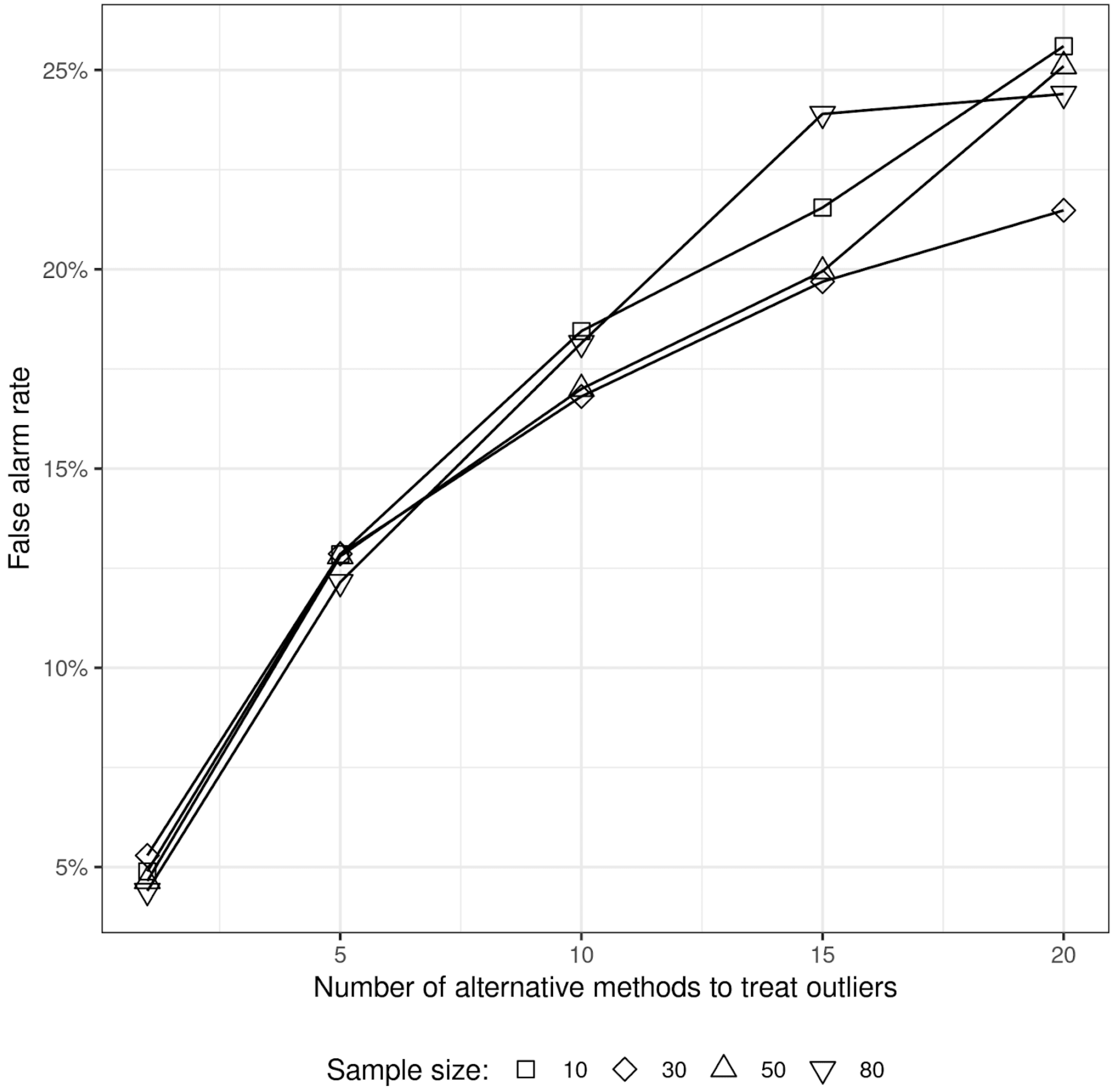

Figure 6 The relationship between sample size and the inflation of false alarm rates.

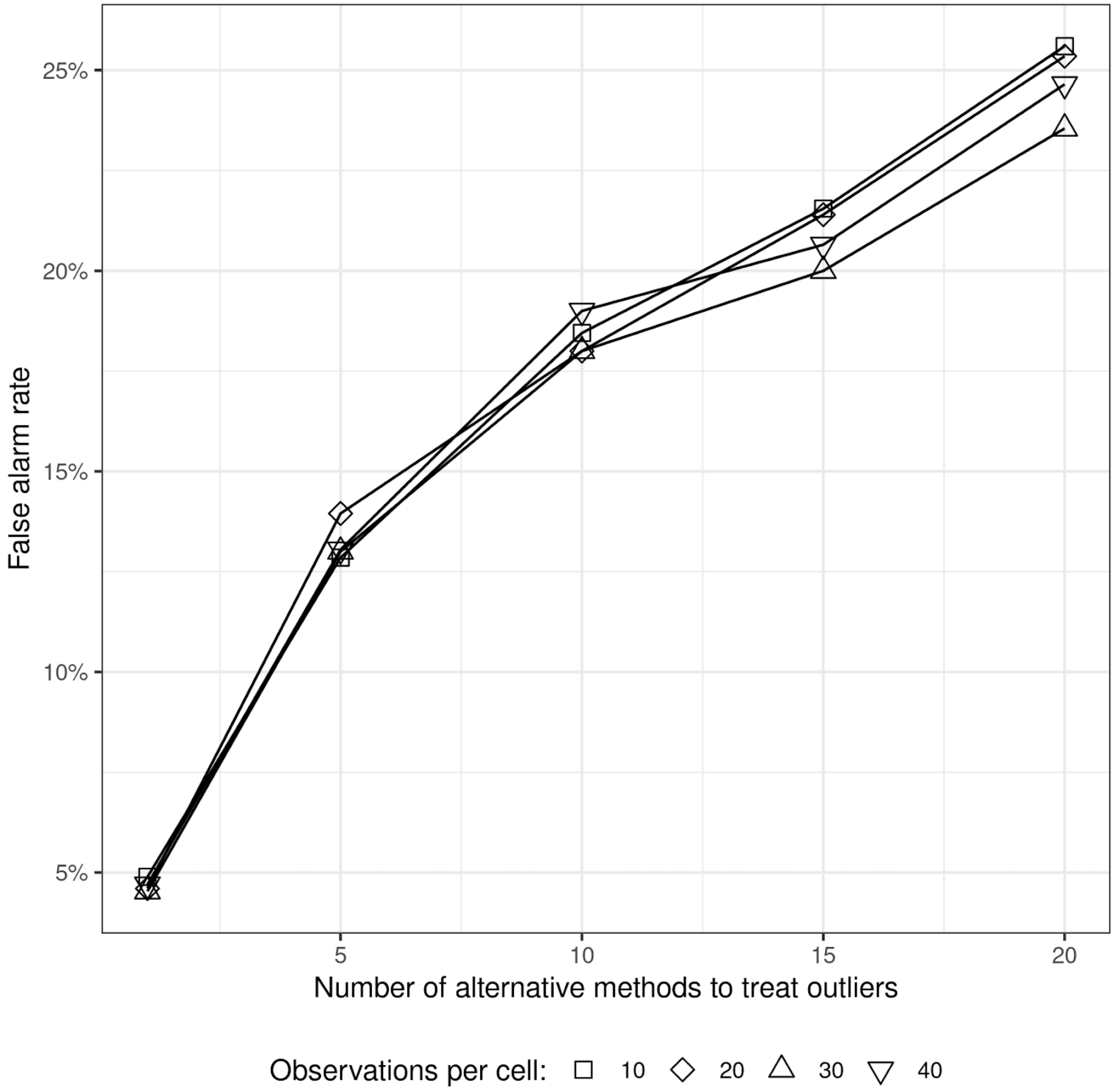

Figure 7 The relationship between the number of observations per cell and the inflation of false alarm rates.
